# Supplementary material for: Stacking Model‐Based Classifiers for Dealing With Multiple Sets of Noisy Labels
Source: Biom J. 2025 Mar 12;67(2):e70042. doi: 10.1002/bimj.70042 (PMC11898607; doi:10.1002/bimj.70042)
Supplement: Supplementary file 1 — Supporting Information [file BIMJ-67-e70042-s001.zip › density-based-ensemble-model-main/competitors_real_data/netlab-master/netlab-master/help/eigdec.htm]

Netlab Reference Manual eigdec


# eigdec

## Purpose

Sorted eigendecomposition

## Synopsis

```
evals = eigdec(x, N)
[evals, evec] = eigdec(x, N)
```

## Description

`evals = eigdec(x, N` computes the largest `N` eigenvalues of the
matrix `x` in descending order. `[evals, evec] = eigdec(x, N)`
also computes the corresponding eigenvectors.

## See Also

`pca`, `ppca`

---

**Pages:**
Index


---

Copyright (c) Ian T Nabney (1996-9)
